# Genome-Wide Analysis Suggests the Relaxed Purifying Selection Affect the Evolution of *WOX* Genes in *Pyrus bretschneideri*, *Prunus persica*, *Prunus mume*, and *Fragaria vesca*

**DOI:** 10.3389/fgene.2017.00078

**Published:** 2017-06-15

**Authors:** Yunpeng Cao, Yahui Han, Dandan Meng, Guohui Li, Dahui Li, Muhammad Abdullah, Qing Jin, Yi Lin, Yongping Cai

**Affiliations:** ^1^School of Life Sciences, Anhui Agricultural UniversityHefei, China; ^2^State Key Laboratory of Tea Plant Biology and Utilization, Anhui Agricultural UniversityHefei, China

**Keywords:** *WOX* genes, phylogenetic analysis, microsynteny, selection, functional divergence

## Abstract

WUSCHEL-related homeobox (WOX) family is one of the largest group of transcription factors (TFs) specifically found in plant kingdom. WOX TFs play an important role in plant development processes and evolutionary novelties. Although the roles of *WOXs* in Arabidopsis and rice have been well-studied, however, little are known about the relationships among the main clades in the molecular evolution of these genes in Rosaceae. Here, we carried out a genome-wide analysis and identified 14, 10, 10, and 9 of *WOX* genes from four Rosaceae species (*Fragaria vesca*, *Prunus persica*, *Prunus mume*, and *Pyrus bretschneideri*, respectively). According to evolutionary analysis, as well as amino acid sequences of their homodomains, these genes were divided into three clades with nine subgroups. Furthermore, due to the conserved structural patterns among these *WOX* genes, it was proposed that there should exist some highly conserved regions of microsynteny in the four Rosaceae species. Moreover, most of *WOX* gene pairs were presented with the conserved orientation among syntenic genome regions. In addition, according to substitution models analysis using PMAL software, no significant positive selection was detected, but type I functional divergence was identified among certain amino acids in WOX protein. These results revealed that the relaxed purifying selection might be the main driving force during the evolution of *WOX* genes in the tested Rosaceae species. Our result will be useful for further precise research on evolution of the *WOX* genes in family Rosaceae.

## Introduction

The WUSCHEL-related homeobox (WOX) gene family encodes a group of plant-specific transcription factors (TFs), which belongs to the homeodomain (HD) TF superfamily (Deveaux et al., 2008; Zhang et al., 2015). There are 15 and 13 members of the *WOX* family in *Arabidopsis thaliana* and rice (*Oryza sativa*) genomes, respectively (Haecker et al., 1991; Graaff et al., 2009; Xin et al., 2010). WOX TFs have been reported to play important role in plant development, such as regulating dynamic balance of stem cell division and differentiation, embryo development, and post-embryonic development (Palovaara et al., 2010; Yadav et al., 2010; Ueda et al., 2011). Bioinformatics analysis showed that *WOX* homology sequences were found in the genomes of *Selaginella*, *Bryophyta* and *Chlorophyta*, but not in the *Rhodophyta* genome, indicating that *WOX* family might originate from green algae (Mukherjee et al., 2009; Lian et al., 2014). According to the phylogenetic analysis among *A. thaliana* and *Petunia hybrida*, tomato (*Solanum lycopersicum*) and rice (*O. sativa*), the *WOX* family was divided into three separate clades: WUS/modern clade, the intermediate clade, and the ancient clade (Haecker et al., 1991). Research on the structural characteristics of WOX members showed that the evolutionary branch members contained a specific WUS box (T-L-X-L-F-P-X-X, where X represents an amino acid) (Haecker et al., 1991). WUS box is an essential component for WUS regulation of stem tip meristem stem cell homeostasis and floral meristem morphogenesis (Ikeda and Ohme-Takagi, 2009). Moreover, *AtWUS* and *AtWOX5* within modern/WUS clade, can redundantly maintain the apical stem cells under undifferentiated status (Sarkar et al., 2007); *AtWOX4* can influent the process of secondary growth by modulating the activity of vascular cambium (Hirakawa et al., 2010); *AtWOX1/3* can coordinate the development of paraxial and distal ends during the leaf development; the primordial initiation and development within meristem were terminated by overexpression of *AtWOX6*. Within the intermediate clade, *AtWOX9* can maintain the growth and division of meristematic cells (Wu et al., 2005); *AtWOX11* was specifically expressed in the cambium, and can promote the formation of adventitious roots (Zhao et al., 2009). Within the ancient clade, *AtWOX13* can promote the formation of embryonic placenta during fruit development (Romera-Branchat et al., 2013); *AtWOX14* and *AtWOX4* can redundantly regulate the differentiation of vascular meristem (Etchells et al., 2013). These studies suggest that the *WOX* gene family is widely involved in the regulation of plant meristem. The members of *WOX* gene family appear to be functionally diverse. Although this gene family in some model plants, such as Arabidopsis and rice, has been studied on a phylogenetic scale, a comprehensive molecular evolutionary study remains elusive in Rosaceae species. Recently, a number of researches on application of comparative genome in analysis of evolution and function of the gene family have been reported (Cao et al., 2016a). Similar to other highly conserved genes, the *WOX* gene and its flanking sequences are likely to be conserved with microsynteny, which can promote the transfer of genetic knowledge among the related species of Rosaceae. The genomes of pear (*Pyrus bretschneideri*), peach (*Prunus persica*), mei (*Prunus mume*), and strawberry (*Fragaria vesca*) were published in Shulaev et al. (2011), Zhang et al. (2012), Verde et al. (2013), and Wu et al. (2013), respectively. Therefore, the availability of whole-genome sequences for four members of three Rosaceae subfamilies (*Fragaria, Prunus*, and *Pyrus*) have enabled us to explore the selection regimes under which *WOX* genes have diversified during the radiation of the Rosaceae. The present research could lead to a better understanding of *WOX* gene family on evolutionary history and diversification in Rosaceae.

## Materials and Methods

### Database Search

*WOX* genes were identified from the genome data representing the four Rosaceae species (*P. bretschneideri*, *P. persica*, *F. vesca*, and *P. mume*, respectively). Two different methods were used to identify *WOX* genes in the *P. bretschneideri*, *P. persica*, *F. vesca*, and *P. mume* genome: (1) BLASTP search using Arabidopsis and rice WOX protein sequences according to previous research methods (Cao et al., 2016a,d), and (2) the screening of Hidden Markov Model profile (PF00046) in four Rosaceae genome using DNAtools software with an e-value cut off of 0.001 (Gehring, 1992). All candidate WOX proteins were confirmed to have a complete WOX domain using both Pfam (Punta et al., 2011), SMART dadabases (Letunic et al., 2012) and InterProScan database (Zdobnov and Apweiler, 2001).

### Phylogenetic Trees Construction

The multiple alignment of WOX proteins in five plant species (*P. bretschneideri*, *P. persica*, *F. vesca*, *P. mume*, and *A. thaliana*) was performed using CLUSTAL_X software (Thompson et al., 1997). Subsequently, we constructed NJ (neighbor-joining) tree using MEGA version 5.1 software (Tamura et al., 2011) with the following parameters: bootstrap (1000 replicate), pairwise deletion and Poisson correction. At the same time, we used ML (maximum-likelihood) and ME (Minimum-evolution) methods to generate the phylogenetic trees to validate the topologies.

### Exon–Intron Structural Analysis and Identification of Conserved Motifs

The online program Gene Structure Display Server (Hu et al., 2014) was used to detect the exon–intron structure of cDNAs and genomic DNA sequences. Subsequently, the MEME (Multiple Em for Motif Elicitation, Version 4.11.1) program (Bailey et al., 2015) was used to obtain the motifs in all candidate WOX proteins, with the parameters: the maximum number of motifs at 20, and the optimum motif width between six and 200 residues. Furthermore, the Pfam database (Punta et al., 2011), SMART software (Letunic et al., 2012), and InterProScan database (Zdobnov and Apweiler, 2001) were used to annotate these structure motifs.

### Microsynteny Analysis

According to the comparisons of the specific regions containing *WOX* genes, we carried out microsynteny analysis across the four Rosaceae species. Similarly, the *WOX* genes of *P. bretschneideri*, *P. persica*, *P. mume*, and *F. vesca* were categorized based on their classification in the evolutionary tree. Subsequently, all *WOX* genes in *P. bretschneideri*, *P. persica*, *F. vesca*, and *P. mume* were set as anchor sites, according to their physical location. Then the flanking protein-coding genes of the *WOX* gene in one species were compared with those in other species. The criterion for dividing an interspecific synteny block is to locate three or more conserved homologous genes within 100 KB between genomes (BLASTP E-value < 10^-10^) (Wang et al., 2012).

### Selective Pressure and Functional Divergence Analysis

To future understand whether the *WOX* genes have undergone positive selection during evolution, maximum likelihood codon models (site models and branch-site models) in PAML software (Yang, 2007) were performed. Three pairs of models (M0 vs. M3, M1a vs. M2a, and M7 vs. M8) were utilized to detect positive selection sites. In the free site models, M0 (one ratio), M1a (neutral), M2a (selection), M3 (discrete), M7 (beta), and M8 (beta and ω) were evaluated by the likelihood ratio test (LRT). The LRT was used to judge which model was more suitable in the two models, and the amino acids sites with positive selection were obtained by the Bayesian method of PAML software (Yang, 2007).

Functional divergence analysis of amino acid sequence data was performed using Diverge 2.0 combined with constructed phylogenetic tree (Gu, 1999, 2006; Gu et al., 2013). The type I functional divergence led to a change of functional limitation, which was highly correlated with the evolution rate after gene duplication (Gu, 1999, 2006; Gu et al., 2013). Type II functional divergence did not result in a change in the functional limitation of the members after gene duplication, but the change of physical and chemical properties of amino acid residues (Gu, 1999, 2006; Gu et al., 2013).

### *cis*-Acting Elements Analysis

To identify putative *cis*-elements in promoter regions of *WOX* genes, the PlantCARE database (Lescot et al., 2002) was used. 2000 bp genomic sequence upstream of the start codon (ATG) was used for *cis*-acting elements analysis.

### Expression Profiles of *FvWOX* Genes

The normalized data (Fragments Per Kilobase Exon model per Million mapped fragments, FPKM) during *F. vesca* development was reported by Darwish et al. (2013), and available from SGR GBrowse. A gene was thought to be expressed if the FPKM value was greater than or equal to 0 FPKM in at least one of the 14 tissues. Subsequently, the transcriptome data of *FvWOXs* was visualized using the R software^1^.

## Results

### Identification and Chromosomal Distribution of *WOX* Genes in Rosaceae

**FIGURE 1:**
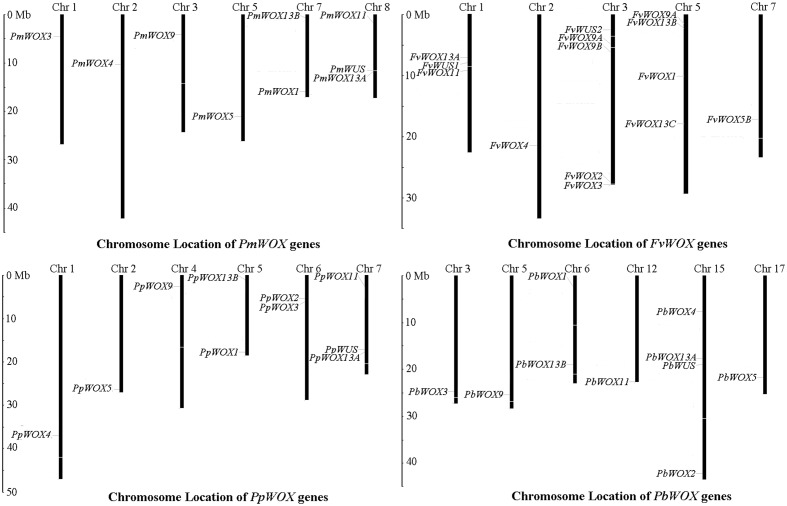
Chromosomal location of *WOX* genes from *Pyrus bretschneideri*, *Prunus persica*, *Fragaria vesca* and *Prunus mume*. The chromosome number was represented by the top of each chromosome.

For identification of *WOXs* gene families, the genome data of tested Rosaceae species was subjected to HMM and BLASTP searches. As a result it is revealed, presence of 9, 10, 10, and 14 *WOX* genes in *P. mume, P. bretschneideri*, *P. persica*, and *F. vesca*, respectively (Supplementary Table S1). These *WOX* genes were named according to method of Haecker et al. (1991). For this purpose, the phylogenetic tree was carried out based on multiple sequence alignments for the full-length WOX protein sequences of tested Rosaceae species and *A. thaliana*. The identified *WOX* genes of these tested four Rosaceae species were renamed according to the evolutionary relationship as shown in Supplementary Table S1. Subsequently, the distribution of these *WOX* genes on chromosomes was identified, based on genomic annotation information. As shown in **Figure 1**, it is discovered that the *WOX* genes were unevenly distributed among the chromosomes in each of tested four species. In the *P. bretschneideri* genome, four of *WOX* genes were distributed on chromosome 15, while remaining distributed on chromosomes 3, 5, 6, 12, and 17. In *F. vesca*, five and four of 14 *WOX* genes were distributed among chromosomes 3 and 5, respectively. In both *P. persica* and *P. mume*, three *WOX* genes distributed were found on one chromosome (no. 7 and no. 8, respectively), with others scattered across different chromosomes (**Figure 1**).

### Evolution of *WOX* Genes in Rosaceae

**FIGURE 2:**
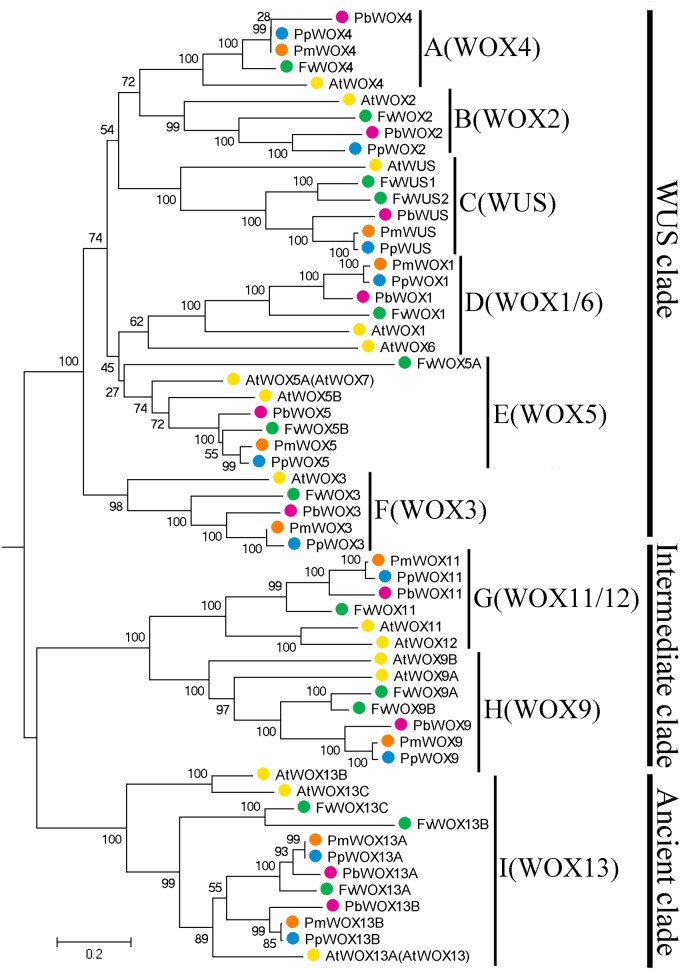
Neighbor-Joining tree of *WOX* family members in four Rosaceae species, *F. vesca* (Fv, green), *P. mume* (Pm, orange), *P. persica* (Pp, blue), and *P. bretschneideri* (Pb, red). Numbers indicate bootstrap support for branches. The clade I *WOX* genes (and only this group) are found both in some green algae and in all land plants, and so provide a root for this tree.

To investigate the possible evolutionary history of the *WOX* genes in the tested Rosaceae species, we carried out a joint phylogenetic analysis using three methods; ME, ML, and NJ. Based on previous report that WOX13 subfamily was an ancient member in the *WOX* gene family (Deveaux et al., 2008), the WOX13 subfamily was selected as an outgroup to root phylogenetic tree. All the tree topologies generated by the three methods (ME, ML, and NJ) were largely consistent with each other, with only minor changes in internal branches (**Figure 2** and **Supplementary Figures S1**, **S2**). Therefore, only NJ phylogenetic tree was used in the following analysis. Previous studies on *WOX* genes have confirmed that motifs FYWFQNH, FYWFQNR, and YNWFQNR were representative markers for the WUS/modern clade, intermediate clade and ancient clade, respectively (Graaff et al., 2009; Ge et al., 2016). Confining the previous results (Deveaux et al., 2008; Xin et al., 2010), our evolutionary analysis exposed a total of 58 members of the *WOX* genes in *P. bretschneideri*, *P. persica*, *F. vesca*, *P. mume* along with *A. thaliana.* These 58 members of *WOX* genes were divided into three clades and nine subfamilies. The Modern clade contained a total of six subfamilies (WUS, WOX1, WOX2, WOX3, WOX4, and WOX5), and Intermediate clade included two subfamilies (WOX9 and WOX11), while Ancient clade just had a WOX13 subfamily, which were consistent with the evolutionary relationships of WOXs in other species (Xin et al., 2010; Hedman et al., 2013; Nardmann and Werr, 2013; Lian et al., 2014). Remarkably, we found that all subfamilies contained at least one *WOX* member from each of the four Rosaceae species (**Figure 2**). These results imply that rapid duplication of *WOX* genes occurred before these dicotyledonous species were diverged.

**FIGURE 3:**
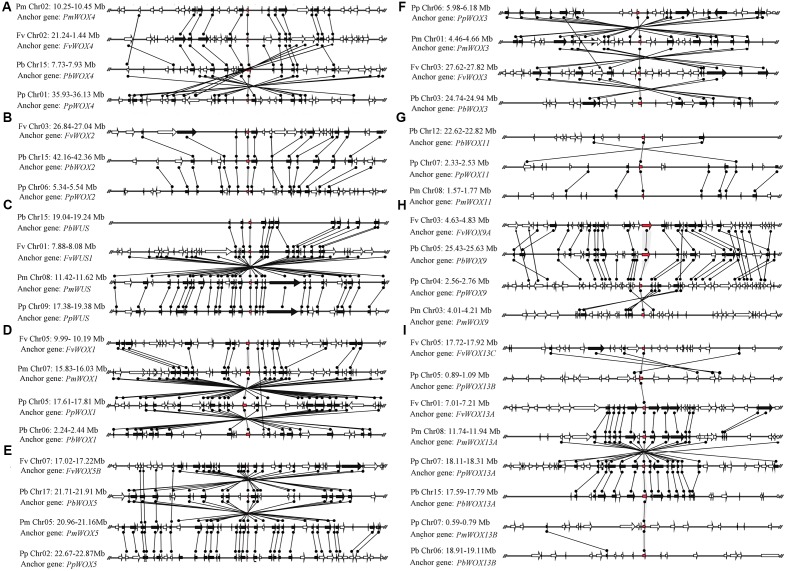
Extensive microsynteny of *WOX* regions across *P. bretschneideri* (Pb), *P. persica* (Pp), *F. vesca* (Fv), and *P. mume* (Fm) chromosomes. The gene’s orientation on strands was indicated by the triangle. Remarkably, the relative positions of all flanking protein-coding genes were defined by anchored *WOX* genes, highlighted in red. Subsequently, we used black lines to connect the homologous genes on two fragments. All genes are numbered from left to right, in order, for each segment. The **(A–I)** subfamilies in figure were consistent with those in **Figure 2**.

Phylogenetic analysis revealed that three pairs of paralogous genes were found among the *WOX* genes, which were consistent with the previous notion that most members of the *WOX* gene family are represented by pairs of orthologous genes. As shown in **Figure 3**, up to eight pairs of orthologous *WOX* genes were shared by *P. persica* and *P. mume*, whereas only two pairs of orthologous *WOX* genes found between *P. bretschneideri* and *P. persica*. However, no orthologous *WOX* genes were found between *F. vesca* and other species. These results were consistent with the evolutionary relationships among these four Rosaceae species (Dickinson et al., 2007; Cao et al., 2016b).

### Analysis of Exon–Intron Structure and the Conserved Motifs

**FIGURE 4:**
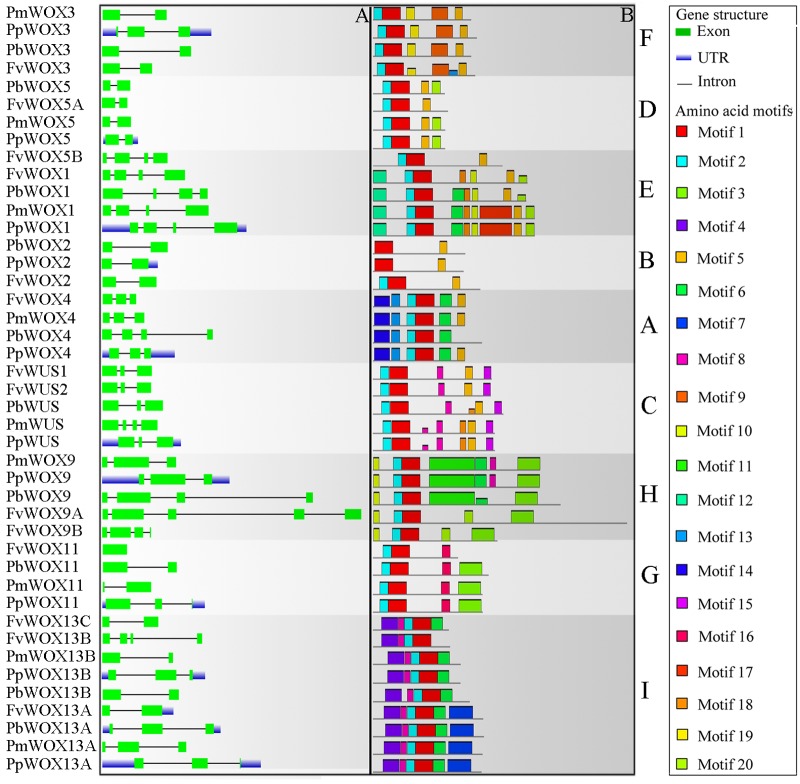
Exon/intron structure **(A)** and motif compositions **(B)** of the Rosaceae *WOX* genes. Relative protein or gene lengths can be estimated by gray bars. Untranslated regions (UTRs), exons and introns are represented by blue lines, green boxes and gray lines, respectively. Motif sequences were shown in Supplementary Table S2. The A-I subfamilies in figure were consistent with those in **Figure 2**.

Previous studies have shown that gene structural diversity is an important resource for the evolution of multigene families (Liu et al., 2009; Cao et al., 2016c). To understand the structural diversity of the *WOX* genes in Rosaceae, gene structures of *PbWOXs*, *PpWOXs*, *PmWOXs*, and *FvWOXs* were deduced. It is revealed that these *WOX* genes contained different numbers of exons as shown in **Figure 4A**. For example, *FvWOX11* only contained one exon, while *FvWOX9A* contained the largest number of exons (5). Moreover, 16, 15, and 3 of *WOX* genes contained two, three and four exons, respectively. These results suggested that the functional diversity of *WOX* genes may be in consequence due to exon loss or gain during the evolution of the *WOX* gene family. Subsequently, gene structures of the *WOX* paralogous and orthologous gene pairs were further analyzed. Among these genes, we found that the exon number of seven gene pairs had changed, including *FvWOX9A*/*FvWOX9B*, *FvWOX13B/FvWOX13C*, *PmWOX3*/*PpWOX3*, *PbWOX4*/*PpWOX4*, *PmWUS*/*PpWUS*, *PmWOX11*/*PpWOX11*, and *PmWOX13B*/*PpWOX13B.* By comparing among these seven gene pairs, it was found that one exon was lost in *FvWOX9B*, *PmWOX3*, *PpWOX4*, *PpWUS*, *PmWOX11* and *PmWOX13B*, while one exon was obtained in *FvWOX9A*, *PpWOX3*, *PbWOX4*, *PmWOXWUS*, *PpWOX11*, and *PpWOX13B.* It may happen during the long evolutionary period. Previous studies have proposed that introns could be specifically inserted and remained in the plant genome during evolution (Rogozin et al., 2003; Carmel et al., 2007; Cao et al., 2016d). In our study, these phenomenon were observed, which might explain the functional differences and diversity of closely related *WOX* genes, such as *PbWOX3* and *FvWOX3*, *PbWOX13B*, and *FvWOX13A* (**Figure 4A**).

Furthermore, it was observed that 20 of the conserved motifs were found in the 43 WOX proteins using MEME website (Supplementary Table S2). These motifs were annotated by using Pfam and SMART. Motif 1, present in all subfamilies, was identified to encode for a conserved homodomain. In addition to the homodomain, most of the WOX members within the same clade shared the similar motif compositions as shown in **Figure 4B**. These results reinforced the classification of WOX subfamilies. However, several motifs were unique to the proteins in some clades. For example, Motif 4 was unique to Ancient clade (clade I: WOX13 subfamily), Motif 5 to WUS clade (clades A–F) and Motif 15 to clade C (Supplementary Table S2 and **Figure 4B**). To some extent, these specific motifs may play an important role in the clade or subfamily, as well as contribution to the functional divergence of *WOX* genes.

### Sequence Analysis of WOX Domains

**FIGURE 5:**
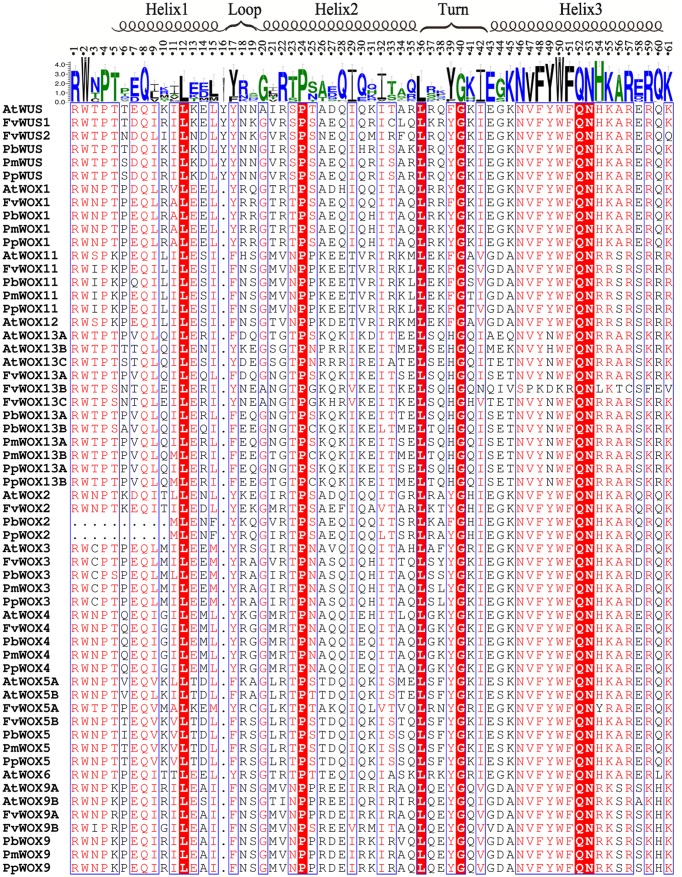
Alignment of the WOX homodomain sequences in five plant species. Highly conserved residues of homodomains were represented by the red shaded blocks in the five tested species. The secondary structure was indicated according to Mayer et al. (1998).

Based on their amino acid sequences, the newly identified *WOX* gene family members were found to contain the conserved homeodomain by multiple sequence alignment of Cluxa2.0 with default parameters. The conserved homeodomain was selected for the visualized results by ESPript 3 (Gouet et al., 2005). The homeodomain structures of these four species were highly similar with each other. They contained a helix-loop-helix-turn-helix structure with either 65 or 66 amino acid residues. A total of 11 conserved sites (Q, L and Y in helix1; I, V, W, F, N, K, and R in helix3) of homeodomain reported previously (Gehring, 1992; Xin et al., 2010), were also conservative in the *WOX* proteins of the Rosaceae species (**Figure 5**). These findings suggested that these amino acid residues could play an important role in their functions. In addition to the previously reported conserved amino acid sites, other conserved amino acid sites have been identified in this study, such as P, L, and I in helix 2, Q and F in helix 3, as well as G in the turn region. Interestingly, an extra Y residue was found in the homeodomain of PbWUS, PmWUS, PpWUS, FvWUS and AtWUS, compared with other members of *WOX* gene family in *P. bretschneideri*, *P. persica*, *P. mume* and *F. vesca* and *A. thaliana*. Similar finding have been reported by Mayer et al. (1998) and Xin et al. (2010), that the homeodomains of *A. thaliana* WUS, *O. sativa* WUS, *Z. mays* WUS1, *Z. mays* WUS2, *S. bicolor* WUS and *P. trichocarpa* WUS were composed of 66 amino acid residues containing an extra Y residue by multiple sequence alignment, which indicates that this residue might play an important role on the function of WUS TF. Remarkably, in Arabidopsis, AtWOX5 (without Y residue between Helix1 and Loop) could replace AtWUS (containing Y residue) to maintain the dynamic balance of stem cells in the shoot apical meristem (Sarkar et al., 2007)

It was reported previously that the WUS protein contains three functional domains, including WUS-box, acidic region and EAR-like motif (Xin et al., 2010; Xiaoxu et al., 2016). These functional domains significantly contribute to its function as a TF (Xin et al., 2010; Xiaoxu et al., 2016). In present study, Motif 5 (WUS box: amino acids, TLLFP) was observed to be in the presence of all WOX proteins in WUX clade (clades A–F) (Supplementary Table S2 and **Figure 4B**). In clade C, the Motif 15 (EAR-like motif: amino acids, SLELSL) was found in all WOX proteins. However, no acidic region was identified in all WOX proteins (Supplementary Table S2 and **Figure 4B**). These results were consistent with previous findings that acidic region may be an important function domain only in Arabidopsis *WUS* gene (Xin et al., 2010; Xiaoxu et al., 2016).

### Microsynteny Analysis of *WOX* Genes

Microsynteny has been surveyed in different species to understand the position of the homologous genes (orthology or paralogy) (Cannon et al., 2003; Yan et al., 2004; Cao et al., 2016a). In this study, microsynteny analysis was carried out for identification of homologous relationships within the *WOX* genes in *P. bretschneideri*, *P. persica*, *F. vesca*, and *P. mume* (**Figure 3**). Additionally, to measure the linkages and molecular history among *WOX* genes, a stepwise gene-by-gene reciprocal comparison was performed. In general, if the flanking genes in the chromosome region of the target gene contained three or more pairs of genes that are collinear, they could be considered as the conserved microsynteny (Lin et al., 2014; Cao et al., 2016a).

Primarily, the intraspecies microsynteny was investigated among four Rosaceae species. However, it was revealed that no collinear *WOX* genes were observed (**Figure 3**). These results suggested that independent duplication events were the main expansion pattern of *WOX* gene family members. Consequently, we analyzed the relationship of the *WOX* genes within each interspecies. The results exposed that the nine clades containing 38 *WOX* genes were found, among which 10 were from *P. persica* and *F. vesca*, 9 from *P. bretschneideri* and *P. mume*, respectively. Then several higher levels of microsynteny found in subfamilies A–G. Among these microsynteny some were remarkably inverted, duplicated such as *PpWOX3*/*PmWOX3*, *PbWOX1*/*PpWOX1*, and *FvWOX13C/PpWOX13B* (**Figure 3**). Usually, genome segments in the same group may evolve from a single sequence, which led to species differentiation (Tripoli et al., 2005; Jing et al., 2016). However, sequence fragments from the same group are considered to be homologous genes, and their genetic evolution resulted in species segregation (Tripoli et al., 2005; Jing et al., 2016). Remarkably, with the construction of the phylogenetic tree, the conservation of microsynteny in different families gradually emerged. Furthermore, some flanking genes were not conserved in each microsyntenic group. Therefore, it was speculated that these new genes were later than this duplication event. Interestingly, several lower levels of microsynteny were also found, such as *PpWOX13B*/*FvWOX13A* and *PbWOX13A*/*PmWOX13B* in clade I, *PbWOX11*/*PpWOX11* and *PpWOX11*/*PmWOX11* in clade H (**Figure 3**). These results strongly suggested that the ancient large-scale duplications could follow by gene rearrangement and loss.

### Analysis of Selection Pressures and Functional Divergence

To investigate whether *WOX* genes have undergone strong selection pressures in the evolution of *WOX* gene family, we used site and branch-site models in the CODEML program of PAML software to detect positive selection sites (Yang, 2007). However, no positive selection was detected among these genes (Supplementary Table S3). These finding imply that relaxed purifying selection might play a major role in the evolution of *WOX* genes. Nardmann and Werr (2013) have shown that the WO*X* gene expansion was resulted from the increased complexity of plant morphology (Nardmann and Werr, 2013). These results proposed that the novel members after gene expansion were retained with partly overlapping expression domains and functions. These results are similar with findings of Nardmann and Werr (2013) who reported that with the relaxed purifying, dosage effects will lead to a selective advantage (Nardmann and Werr, 2013).

For further investigation to comprehend significant differences in selection pressures among WUS clade, intermediate clade and ancient clade, the branch-site models were performed using PAML software. It was exposed that no significant positive selection observed in the different branches of *WOX* gene family (Supplementary Table S4). This result was in contrast to the previous report that some significant positive selection sites were fixed in *WOX* genes of peanut (Wang et al., 2015). Because significant positive selection usually exerts its effects only in few sites and in a short period of evolutionary processes, it is difficult to detect positive selection. Thus, the selected signal could be diluted by the purifying selection (Zhang, 2005). However, as the *WOX* coding regions are highly conserved among members of orthologous families, the absence of strong positive selection was expected in Rosaceae species.

Due to the fact that significant positive selection could only detect a limited number of adaptive selection events, we performed a functional divergence analysis according to method used by Cao et al. (2016a). The DIVERGE software was used to calculate functional divergence of type I or II between gene clades in *WOX* genes with posterior analysis. In general, type I functional divergence usually resulted in a specific amino acid selectivity change, i.e., evolutionary rate change. The type II functional divergence only led to the change of physical and chemical properties of amino acids, which were occurred after gene duplication. In present study, to avoid the emergence of false positives, the sites with a posterior probability Q K > 0.9 were set as the key amino acid sites arising the functional differences according to previous experimentation reported (Yin et al., 2013; Cao et al., 2016a). Our results showed that five key amino acid sites (144, 154, 161, 165, and 166) were identified as type I functional divergence between Ancient and Modern (**Table 1**), while just one key site (152) between Intermediate and Modern (**Table 1**). The chi-square test (x 2) found that the *P*-values of Ancient/Modern and Intermediate/Modern were less than 0.05, reaching a significant level. Interestingly, among these three clades, no specific type II functional divergence site (Q K > 0.9) was detected (Supplementary Table S5), suggesting that the physicochemical properties of amino acid sequences between these Rosaceae *WOX* genes were highly identical.

**Table 1:** Analysis of type I functional divergence.

Group 1	Group 2	Θ ± SE	LRT	Q K > 0.9	*P*
Ancient	Intermediate	0.277 ± 0.286	0.938	Not allowed	*P* < 0.05
Ancient	Modern	0.754 ± 0.153	24.289	144,154,161,165,166	*P* < 0.05
Intermediate	Modern	0.368 ± 0.114	10.454	152	*P* < 0.05

*SE, standard error; LRT, value of likelihood ratio test*.

### *cis*-Acting Element Analysis of *WOX* Genes

Two thousand bp sequences of upstream from start codon (ATG) among the putative *WOX* genes, were used for analysis of *WOX* promoters by searching, against the PlantCARE website. Consequently, we detected various types of *cis*-acting elements in the promoter region of 43 *WOX* genes (Supplementary Table S6). These results indicated that the same type of *WOX* might carry out different functions. MBS and ABRE elements were found to be distributed in promoter region of most *WOX* genes, implying that *WOX* genes were transcriptionally regulated upon salt stress and dehydration. Remarkably, we found that the *cis*-elements exhibit significant differences in the promoter regions of duplicated *WOX* genes. These results indicated that the duplicated *WOX* genes may exhibit different regulation features.

### Expression Profiles of *F. vesca WOX* Genes

To explore the role of the *WOX* gene family in *F. vesca* development process, the expression of the *FvWOX* genes was explored. The results showed that their expression levels were divergent from each other, indicating that they may be functionally active among all tissues except *FvWOX5B* (**Supplementary Figure S3**), which was located in Pollen with no expression. At the same time, most of *FvWOX* genes exhibited developmental stage-specificity, such as higher expression of *FvWOX13A*, *FvWOX3*, *FvWOX13B*, and *FvWOX1* in flowering, and *FvWOX4*, *FvWOX5*, and *FvWOX5A* in embryo (**Supplementary Figure S3**). Surprisingly, we found that *FvWOX13A*, *FvWOX9A*, and *FvWOX1* were highly expressed among all tissues, indicating that these genes were persistent and very important during development process of *F. vesca*.

## Discussion

In present study, 43 *WOX* genes from four Rosaceae species were identified. It is observed that no direct relevance between genome sizes and the number of *WOX* gene family members. For example, there was no significant variety in the genome size of *P. bretschneideri* (271.9 Mb) (Wu et al., 2013) and *F. vesca* (240 Mb) (Shulaev et al., 2011), the number of *WOX* genes have been obviously changed. On the contrary, the number of *WOX* genes of the *P. persica* (224.6 Mb) (Verde et al., 2013) and *P. mume* (201 Mb) (Zhang et al., 2012) had a corresponding relationship with their genome sizes. In addition, we also noted that *P. bretschneideri* undergoes two genome-wide duplication events compared with those from *P. persica*, *P. mume*, and *F. vesca* (Wu et al., 2013). Nevertheless, the members of the *WOX* gene family among these four species did not change significantly. These findings indicate that the recent genome-wide duplication event did not contribute to the expansion of *P. bretschneideri WOX* gene family numbers. These results were supported by microsynteny analysis (**Figure 3**).

Previous studies suggested that the *WOX* gene family was divided into three major clades; the ancient clade were mainly present in land plants and green algae, while the intermediate and modern clades were only present in ferns and seed plants (Deveaux et al., 2008; Graaff et al., 2009; Nardmann and Werr, 2012, 2013). In present study, we found that all *WOX* genes from four Rosaceae species were distributed in the three clades, and was supported by the result of exon–intron and conserved domains analysis. At the same time, we also found that each clade contained its specific conserved motifs, implying these specific conserved motifs were likely required for subfamily-specific functions, such as Motif 5 to WUS clade (clades A–F). In the *WOX* gene family, the modern/WUS clade and intermediate clade were evolved from the ancient clade. It is well-known that gene sequence divergence, recombination, and duplications were considered to be the main driving forces for the evolution of gene families (Lin et al., 2014). In our study, the selection pressure was analyzed by using PAML program (Yang, 2007). In general, values of dn/ds (ω) >1, =1, and <1 represents positive selection, neutral evolution and purifying selection on the target gene, respectively. In this study, we found that the ω value of *WOX* genes was 0.07304 in M0 model (Supplementary Table S3). These results implied that *WOX* genes from four Rosaceae mainly underwent purifying selection during evolution, which was consistent with the hypothesis that highly conserved genes remain in the genome due to purifying selection. For example, the conserved *WOX* clade genes were all retained from green alga to seed plants (Nei, 2007).

Hedman et al. (2013) found that most conifer *Picea abies WOX* genes expressed at high levels in all developmental stages, while a few *PaWOXs* expression were low in specific tissues (Hedman et al., 2013). Zhang et al. (2015) reported that 10 *Citrullus lanatus WOX* genes were expressed in almost all tissues (Na et al., 2015). In our study, we found that the most of the *FvWOX* genes were expressed in different tissues. Among them, *FvWOX4*, *FvWOX5*, *FvWOX5A*, and *FvWOX9B* were mainly expressed in embryo stage with a very low expression for these genes in other tissues, which implied that these genes might have the same function as the key regulation factor *AtWOX9A* and *AtWOX9B* which was involved in the maintenance of the SAM (Wu et al., 2005; Skylar et al., 2010). The high expression of *FvWOX13A, FvWOX13B*, and *FvWOX13C* (Ancient clade) in flower tissue implied it had an important role similar to *AtWOX13A* and *AtWOX13B* in floral transition (Deveaux et al., 2008).

In this work, we identified 43 *WOX* genes in four Rosaceae species. These genes were divided into three well-supported clades (ancient, modern/WUS, intermediate) with nine subgroups. We also found that *WOX* genes phylogenetic relationship was supported by the presence of gene structure and conserved motif distribution. Our study demonstrated the existence of extensive microsynteny between *WOX* genes by comparing the *WOX* genes across four Rosaceae genomic sequences. The results showed that the maintenance of gene copy number after a whole genome duplication event was the main force to shape the *WOX* family evolution, with the purifying selection and a period of possibly relaxed constraint. Functional divergence was detected among the ancient, intermediate, and modern clades, which leaded to functional constraints, especially different evolutionary rates, after gene duplication. Furthermore, the expression profile of *FvWOX* gene identified that these genes play crucial roles in the floral transition during strawberry growth and development. The comprehensive analysis of the *WOX* family genes and the preliminary results presented here will be useful in the selection of appropriate candidate genes for further research on biological functions of *WOX* genes in strawberry.

## Author Contributions

YuC and YH conceived and designed the experiments; YuC, QJ, and YH performed the experiments; YuC, YH, and DM analyzed the data; YuC, YH, DL, GL, MA, YL, and YoC contributed reagents/materials/analysis tools; YuC and YH wrote the paper.

## Conflict of Interest Statement

The authors declare that the research was conducted in the absence of any commercial or financial relationships that could be construed as a potential conflict of interest.

## Footnotes

1 http://www.bioconductor.org

## Supplementary Material

FIGURE S1 Maximum-Likelihood tree of *WOX* family members in four Rosaceae species.

FIGURE S2 Minimum-Evolution tree of *WOX* family members in four Rosaceae species.

FIGURE S3 Expression patterns of *FvWOX* genes during strawberry growth and development.
