# Supplementary figures and images for: Genome-Wide Analysis Suggests the Relaxed Purifying Selection Affect the Evolution of WOX Genes in Pyrus bretschneideri, Prunus persica, Prunus mume, and Fragaria vesca
Source: Front Genet. 2017 Jun 15;8:78. doi: 10.3389/fgene.2017.00078 (PMC5471313; doi:10.3389/fgene.2017.00078)

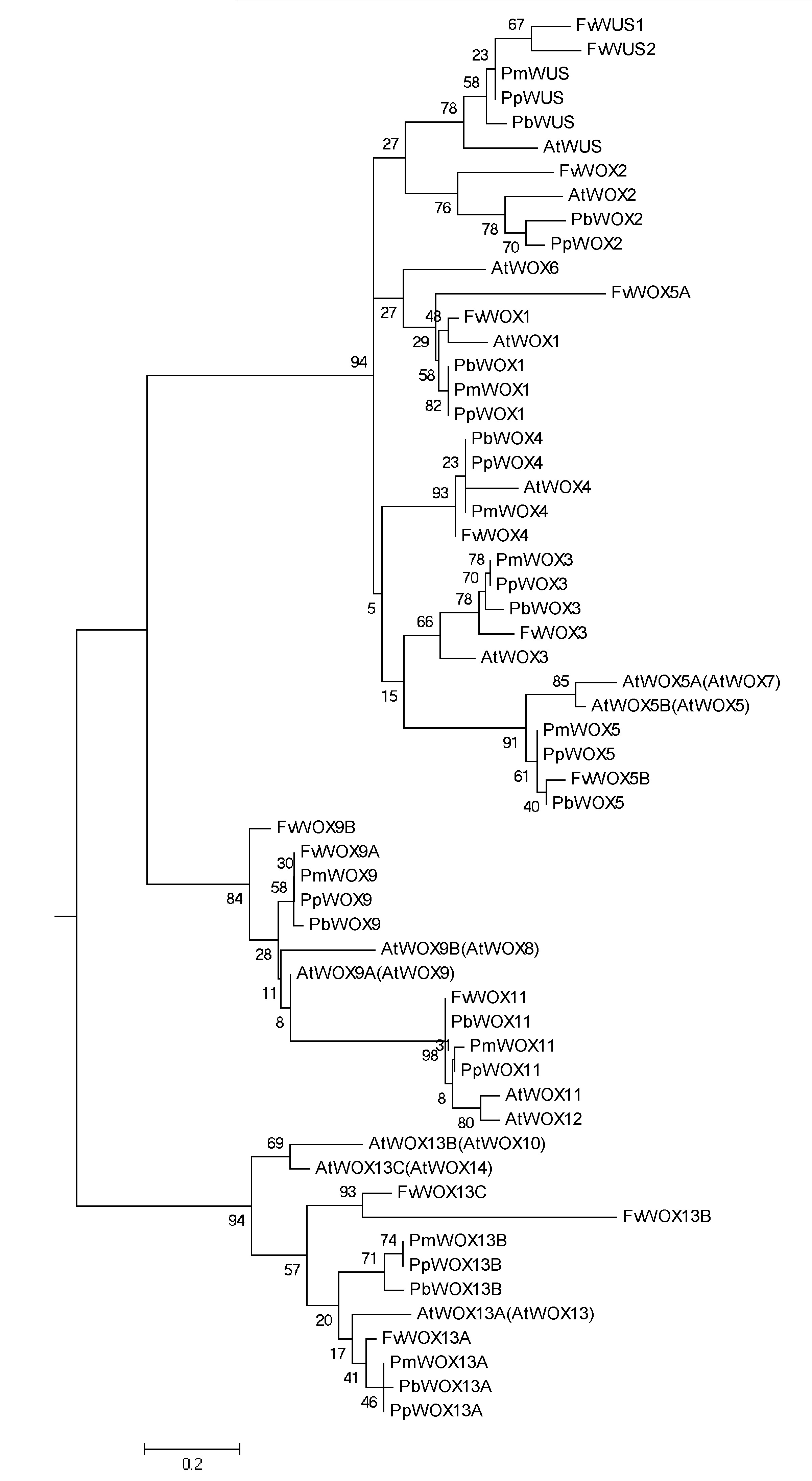

Supplement: FIGURE S1 — Maximum-Likelihood tree of WOX family members in four Rosaceae species. [file Image_1.TIF]

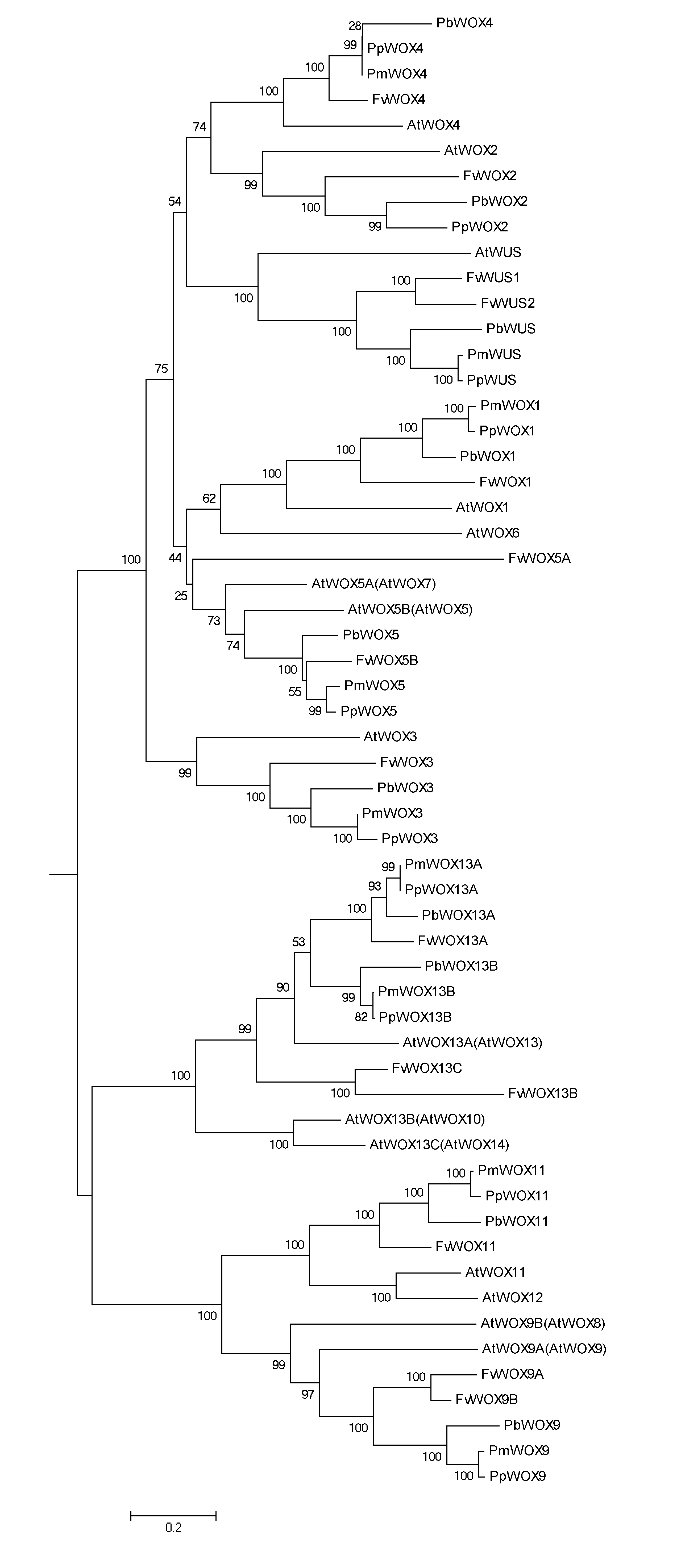

Supplement: FIGURE S2 — Minimum-Evolution tree of WOX family members in four Rosaceae species. [file Image_2.TIF]

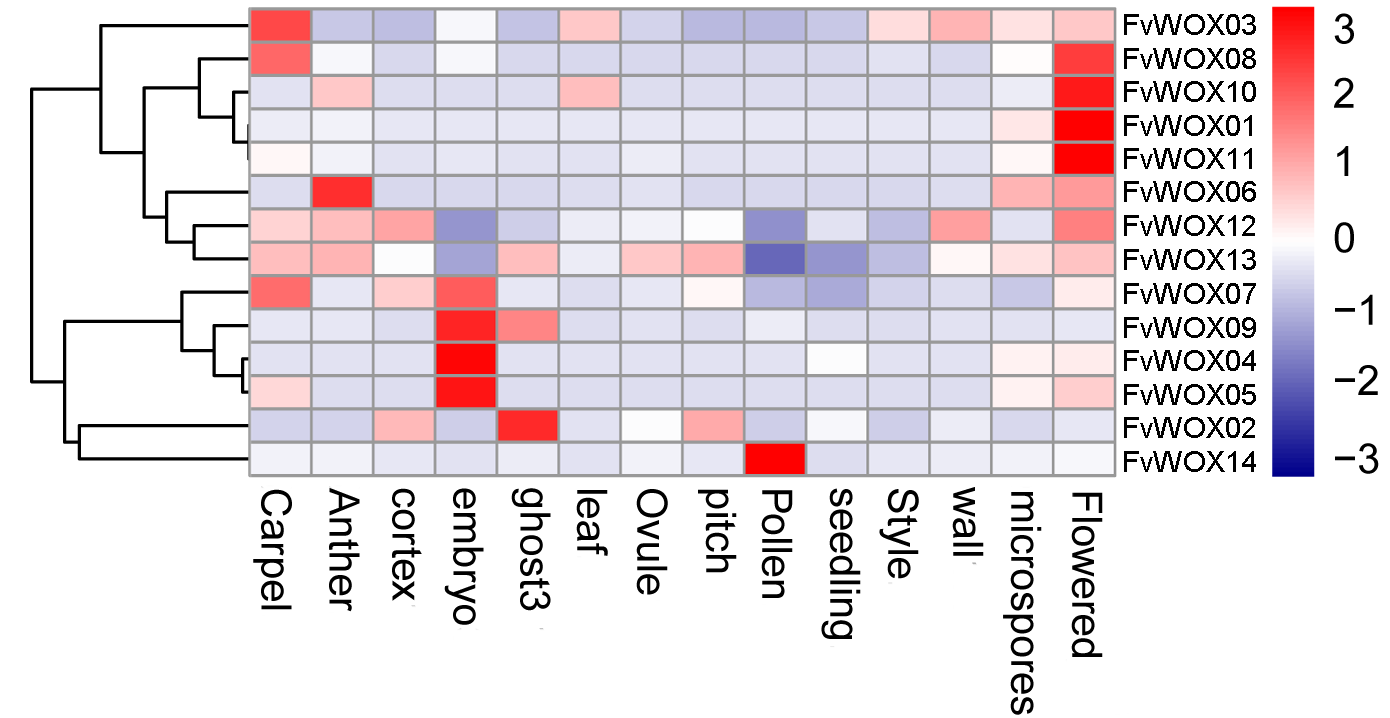

Supplement: FIGURE S3 — Expression patterns of FvWOX genes during strawberry growth and development. [file Image_3.TIF]
