# Supplementary material for: Genome-Wide Analysis Suggests the Relaxed Purifying Selection Affect the Evolution of WOX Genes in Pyrus bretschneideri, Prunus persica, Prunus mume, and Fragaria vesca
Source: Front Genet. 2017 Jun 15;8:78. doi: 10.3389/fgene.2017.00078 (PMC5471313; doi:10.3389/fgene.2017.00078)
Supplement: Supplementary file 4 [file Table_1.DOCX]

The detailed information of *WOX* genes from pear, peach, yang mei and strawberry.

| **Gene Name** | **Gene Identifier** | **Chromosme** | **5′ End** | **3′ End** |
| --- | --- | --- | --- | --- |
| FvWOX3 | mrna14025.1 | chr3 | 27718000 | 27719297 |
| FvWOX4 | mrna09136.1 | chr2 | 21341222 | 21342100 |
| FvWOX1 | mrna09389.1 | chr5 | 10088689 | 10090852 |
| FvWOX5B | mrna20925.1 | chr7 | 17123250 | 17123919 |
| FvWOX2 | mrna14133.1 | chr3 | 26942002 | 26943422 |
| FvWUS1 | mrna14621.1 | chr1 | 7976321 | 7977629 |
| FvWOX9A | mrna29786.1 | chr3 | 4733097 | 4739905 |
| FvWOX9B | mrna28935.1 | chr3 | 6059292 | 6060577 |
| FvWOX11 | mrna30337.1 | chr1 | 9154081 | 9154719 |
| FvWOX5A | mrna32456.1 | chr5 | 1437335 | 1439039 |
| FvWOX05 | mrna30464.1 | chr3 | 2519705 | 2520993 |
| FvWOX13A | mrna13035.1 | chr1 | 7106141 | 7108115 |
| FvWOX13C | mrna20491.1 | chr5 | 17819758 | 17821231 |
| FvWOX13B | mrna31986.1 | chr5 | 2185127 | 2187742 |
| PbWOX4 | Pbr0196681 | Chr15 | 7730703 | 7733577 |
| PbWOX3 | Pbr0339531 | Chr3 | 24746888 | 24749198 |
| PbWOX1 | Pbr0283831 | Chr6 | 2240127 | 2242845 |
| PbWUS | Pbr0221151 | Chr15 | 19043970 | 19045533 |
| PbWOX5 | Pbr0113391 | Chr17 | 21710763 | 21711474 |
| PbWOX9 | Pbr0004321 | Chr5 | 25434379 | 25439878 |
| PbWOX11 | Pbr0355141 | Chr12 | 22623974 | 22625890 |
| PbWOX13A | Pbr0373251 | Chr15 | 17593570 | 17596629 |
| PbWOX13B | Pbr0067732 | Chr6 | 18917061 | 18919037 |
| PbWOX2 | Pbr0208331 | Chr15 | 42165923 | 42167610 |
| PmWOX3 | Pm000753 | chr1 | 4562403 | 4564029 |
| PmWOX4 | Pm005311 | chr2 | 10350782 | 10351836 |
| PmWOX1 | Pm025084 | chr7 | 15927902 | 15930588 |
| PmWOX5 | Pm018890 | chr5 | 21055588 | 21056313 |
| PmWUS | Pm026748 | chr8 | 11524297 | 11525701 |
| PmWOX9 | Pm010370 | chr3 | 4113018 | 4114900 |
| PmWOX11 | Pm025458 | chr8 | 1672715 | 1673942 |
| PmWOX13B | Pm022951 | chr7 | 692219 | 694017 |
| PmWOX13A | Pm026793 | chr8 | 11838088 | 11840222 |
| PpWOX3 | Prupe.6G088900.1.p | chr06 | 6087660 | 6090438 |
| PpWOX4 | Prupe.1G432100.1.p | chr01 | 37002438 | 37004290 |
| PpWOX1 | Prupe.5G232600.1.p | chr05 | 17700766 | 17704458 |
| PpWOX5 | Prupe.2G247100.1.p | chr02 | 26343294 | 26344183 |
| PpWUS | Prupe.7G167700.1.p | chr07 | 17082135 | 17084145 |
| PpWOX9 | Prupe.4G055300.1.p | chr04 | 2655776 | 2659034 |
| PpWOX11 | Prupe.7G016600.1.p | chr07 | 2402515 | 2405136 |
| PpWOX13A | Prupe.7G183300.1.p | chr07 | 17805446 | 17808091 |
| PpWOX13B | Prupe.5G009600.1.p | chr05 | 990843 | 994898 |
| PpWOX2 | Prupe.6G080100.1.p | chr06 | 5453740 | 5455174 |
