# Supplementary material for: Genome-Wide Analysis Suggests the Relaxed Purifying Selection Affect the Evolution of WOX Genes in Pyrus bretschneideri, Prunus persica, Prunus mume, and Fragaria vesca
Source: Front Genet. 2017 Jun 15;8:78. doi: 10.3389/fgene.2017.00078 (PMC5471313; doi:10.3389/fgene.2017.00078)
Supplement: Supplementary file 5 [file Table_2.DOCX]

| Major MEME motif sequences in WOX proteins from *P.bretschneideri*, *P. persica*, *P.mume* and *F. vesca*. | | | |
| --- | --- | --- | --- |
| Motif | Best possible match | Best possible match | |
| 1 | 46 | IYRSGMRTPSAEQIQHITAQLRKYGKIEGKNVFYWFQNHKARERQK | |
| 2 | 21 | HCITVCTRWCPTPEQIRILED | |
| 3 | 56 | VFINDVAFEVPAGPFNVREAFGDDAVLIHSSGHPVLTNEWGFTLHSLHHGAFYYLV | |
| 4 | 41 | YVKVMTDEQMETLRKQIAVYATICEQLVEMHKALTAQQDLA | |
| 5 | 19 | EPCCEIETLQLFPLHSEDI | |
| 6 | 29 | QWNPACNCCESEVEREVDSPYDKKTRPWD | |
| 7 | 59 | SAQRTEDLCFQSPEISSDLHFLDPQTTKTDKMFPSNSGLRTSRHLSEMSFYDEVLSNSR | |
| 8 | 15 | MRMGNFYCDCLMTSG | |
| 9 | 41 | PQVGVEDALMNHAWKMEIPQRVEMEKSVMSMYGRDWMMMMM | |
| 10 | 15 | AERNRHWPMMFKSCP | |
| 11 | 113 | PVSTSTLNIINAAAPSSSSSSSEKSSPKSAPNNKAFSMGFSSNVAAHDVSINSPTASVNQQRSFFQPHHQHHSEILPEPFFFPMHPNPNCNFTQGFCFSELSNVVDVPNHGTH | |
| 12 | 33 | MWMMGYNESGHADFNMPDSFNGRKLRPLIPRPL | |
| 13 | 21 | TTALAPFDLKSFIRPESGPRK | |
| 14 | 39 | SSMKVHQFARGLWEHEPSLTLGCTKRLRPLAPKLANTTA | |
| 15 | 18 | RTSLELSLNSYGHMTPDY | |
| 16 | 21 | DQFFSVSGQMGFPEIEQGSGV | |
| 17 | 80 | LPPQYPHPPTHLIINTPACSTANSNSTDNLTSSRASRAATVRTMDQRLIKGHDLSILLTPYSSISEESGFINFNHPNHNT | |
| 18 | 15 | CRTDGWIQFDEEELQ | |
| 19 | 21 | LYNHHHNYHNYHNNPYHHHHH | |
| 20 | 21 | HANAYFKEAQAFYFSELAEMD | |
