# Supplementary material for: Genome-Wide Analysis Suggests the Relaxed Purifying Selection Affect the Evolution of WOX Genes in Pyrus bretschneideri, Prunus persica, Prunus mume, and Fragaria vesca
Source: Front Genet. 2017 Jun 15;8:78. doi: 10.3389/fgene.2017.00078 (PMC5471313; doi:10.3389/fgene.2017.00078)
Supplement: Supplementary file 6 [file Table_3.DOCX]

Parameter estimates and log-likelihood values under models of variable ω ratios among sites

| **Model** | **Ln L** | **Estimates of parameters** | | | | **LRT pairs** | **p value** | **Positive sites** |
| --- | --- | --- | --- | --- | --- | --- | --- | --- |
| M3 | -3247.991855 | p: | 0.66504 | 0.20595 | 0.12900 | M0/M3 | <<0.001 | No |
|  |  | ω: | 0.02557 | 0.10410 | 0.40236 |  |  |  |
| M0 | 0 | ω0: | 0.07304 | | |  |  | Not Allowed |
| M2a | -3291.437812 | p: | 0.87055 | 0.06259 | 0.06686 | M1a/M2a | 1.000 | No |
|  |  | ω: | 0.06176 | 1.00000 | 1.00000 |  |  |  |
| M1a | -3291.437812 | p: | 0.87055 | 0.12945 |  |  |  | Not Allowed |
|  |  | ω: | 0.06176 | 1.00000 |  |  |  |  |
| M8 | -3264.361432 | p0=0.98015 | p=0.40368 | q=3.52175 |  | M7/M8 | <0.68 | No |
|  |  | (p1=0.01985) | ω= 1.00000 |  |  |  |  |  |
| M7 | -3264.743106 | p= | 0.33524 | q= | 2.63660 |  |  | Not Allowed |
