# Supplementary material for: Genome-Wide Analysis Suggests the Relaxed Purifying Selection Affect the Evolution of WOX Genes in Pyrus bretschneideri, Prunus persica, Prunus mume, and Fragaria vesca
Source: Front Genet. 2017 Jun 15;8:78. doi: 10.3389/fgene.2017.00078 (PMC5471313; doi:10.3389/fgene.2017.00078)
Supplement: Supplementary file 7 [file Table_4.DOCX]

| **Table S4** Parameter estimates and likelihood scores for branch–site models in CodeML | | | | |
| --- | --- | --- | --- | --- |
| Model | Estimates of parameters | lnL | p LRT^a^ | Positively selected sites BEB^b^ (%) |
| Ancient clade as foreground | | | | |
| A_ω=1_ | p_0_: 0.80420 p_1_: 0.11976 p_2a_: 0.06618 p_2b_: 0.00986  background: ω_0_: 0.06154 ω_1_: 1.00000 ω_2a_: 0.06154 ω_2b_: 1.00000  foreground: ω_0_: 0.06154 ω_1_: 1.00000 ω_2a_: 10.12982 ω_2b_: 10.12982 | -3290.549324 | P<0.50 | 4 E 0.779,10 V 0.509,32 E 0.897 |
| Intermediate clade as foreground | | | | |
| I_ω=1_ | p_0_:0.81776 p_1_:0.12159 p_2a_:0.05280 p_2b_:0.00785  background: ω_0_:0.06192 ω_1_:1.00000 ω_2a_:0.06192 ω_2b_:1.00000  foreground: ω_0_:0.06192 ω_1_:1.00000 ω_2a_:10.48495 ω_2b_:10.48495 | -3291.005041 | p<0.60 | 14 N 0.873 |
| Modern clade as foreground | | | | |
| M_ω=1_ | p_0_:o.74546 p_1_:0.11103 p_2a_:0.12491 p_2b_: 0.01860  background: ω_0_:0.06288 ω_1_:1.00000 ω_2a_:0.06288 ω_2b_:1.00000  foreground: ω_0_:0.06288 ω_1_:1.00000 ω_2a_:9.51076 ω_2b_:9.51076 | -3289.694770 | p<0.41 | 5 M 0.527,11 R 0.508,15 A 0.931,34 K 0.613,43 H 0.659,47 D 0.915 |
