# Supplementary material for: Genome-Wide Analysis Suggests the Relaxed Purifying Selection Affect the Evolution of WOX Genes in Pyrus bretschneideri, Prunus persica, Prunus mume, and Fragaria vesca
Source: Front Genet. 2017 Jun 15;8:78. doi: 10.3389/fgene.2017.00078 (PMC5471313; doi:10.3389/fgene.2017.00078)
Supplement: Supplementary file 8 [file Table_5.DOCX]

Analysis of type II functional divergence.

| Group 1 | Group 2 | Θ±SE | Q _k_ >0.9 |
| --- | --- | --- | --- |
| Ancient | Intermediate | 0.235±0.250 | Not allowed |
| Ancient | Modern | 0.460±0.208 | Not allowed |
| Intermediate | Modern | 0.147±0.196 | Not allowed |
